# A case study of binary outcome data extraction across three systematic reviews of hip arthroplasty: errors and differences of selection

**DOI:** 10.1186/1756-0500-6-539

**Published:** 2013-12-17

**Authors:** Christopher Carroll, Alison Scope, Eva Kaltenthaler

**Affiliations:** 1 Health Economics and Decision Science (HEDS), School of Health and Related Research (ScHARR), Regent court, Regent Street, Sheffield S1 4DA, UK

**Keywords:** Systematic review, Data interpretation, Meta-analysis, Data extraction, Data-handling error, Reporting

## Abstract

**Background:**

Data extraction is a key stage in systematic review, yet it is the subject of little research. The aim of the present research was to use a small case study to highlight some important issues affecting this fundamental process.

**Methods:**

The authors undertook an analysis of differences in the binary event data extracted and analysed by three systematic reviews on the same topic: a comparison of total hip arthroplasty and hemiarthroplasty. The following binary event data were extracted for three key outcomes, common to all three reviews, from those trials common to all three reviews: Dislocation rates, 1-year mortality, and revision rates. Differences between the data extracted by the three reviews were categorised as either errors or an issue of data selection. Meta-analysis was performed to assess whether these differences led to differences in summary estimates of effect.

**Results:**

Across the three outcomes, differences in selection accounted for between 8% and 42% of the data differences between reviews, and errors accounted for between 8% and 17%. No rationale was given in any of these former cases for the choice of event data being reported. These differences did lead to small differences in meta-analysed relative risks between the two treatments in the three reviews, but none was significant.

**Conclusions:**

Systematic reviewers should use double-data extraction to minimise error and also make every effort to clarify or explain their choice of data, within the scope of their publication. Reviewers frequently exercise selection when faced with a choice of alternative but potentially equally appropriate data for an outcome. However, this selection is rarely made clear by review authors. Systematic review was developed as a method specifically to be both reproducible and transparent. This case study suggests that neither objective is always being achieved.

## Background

Data extraction or abstraction is a crucial stage in systematic review. It is the stage that generates the data to be analysed. However, it is a relatively under-researched area of the systematic review process compared to information retrieval, the assessment of bias, and methods of synthesis. The little research that has been conducted has been fundamental in our understanding of some of the principal limitations affecting the process. It has found that errors in data extraction can occur frequently. One study found errors in 20 of the 34 Cochrane reviews assessed, or error rates as high as 31% in one evaluation of data extraction [1]. Data extraction errors may occur in all variables extracted for a review, but outcomes appear to generate the most errors: such error rates have been found to be as high as 77% [2]. Errors have been defined in various ways in these studies, principally as inaccuracies, omissions, inadequacies and incomplete data [1,2]. Previous assessments have covered all fields in data extraction, from design and inclusion criteria to actual outcomes. Error rates are apparently unaffected by reviewer experience [1], but can be influenced to a small degree by the double data extraction process [2]: that is, double data extraction can lead to fewer errors, though reviewer experience itself does not. Wherever it has been evaluated, it has been found that the impact of these errors on key outcomes was not statistically significant.

The aim of the present work is to contribute to the small amount of literature on this topic, which is actually a time-consuming and crucial stage in the systematic review process. In doing so it focuses only on key numerical outcome data actually employed in a meta-analysis. This approach was taken because these outcome data are more likely to affect synthesis and findings than other data extracted from a study, and because numerical data potentially present less ambiguity than textual data, such as inclusion or exclusion criteria or descriptions of measures. Previous studies have reported that error rates are lower when the variable is “simple”, such as authorship, country of study, gender distribution and numbers enrolled, where-as text variables, which can sometimes be lengthy, such as inclusion and outcome assessment criteria, prove more problematic [1,2]. Indeed, it has been found that when data handling is particularly complex, for example calculating standardised mean differences (SMDs), in order to permit meta-analysis of different scales using continuous data, the scope for error is increased. [3] In the case of simple binary numerical outcome data, however, the data extracted by two reviewers are quite simply either the same or different. There is little or no scope for variation. Any differences may therefore be explained in two ways. First, they are “errors” over which there is no question: one number alone is correct; any others are an error. Second, differences in the data might be explained by a factor otherwise only rarely considered by the research on this topic [3], that a publication might offer a number of equally viable data alternatives for extraction. In other words, more than one possible answer exists, but reviewers have to make a choice about which number they considered to be the most appropriate.

This research therefore aimed first to identify differences in the binary event data extracted and analysed for three outcomes by three reviews on the same topic, across trials common to all three reviews. The objective was then to determine whether any such differences were due either to reviewers simply choosing different, potentially viable data from the primary study publications, or to outright errors of extraction. The impact of these differences on final outcomes was also evaluated. Finally, the implications of these findings for the implementation of systematic review methods, and the peer-review of systematic reviews, is considered.

## Methods

The authors conducted a systematic review of clinical effectiveness comparing total hip replacement versus hemiarthroplasty for the treatment of displaced intracapsular hip fracture. This is published as a Health Technology Assessment (HTA) [4]. An updated Cochrane review [5] and a British Medical Journal [BMJ] systematic review [6] addressing a very similar question, and applying very similar or the same inclusion criteria, were published shortly before the authors’ own review. The authors’ own HTA report, and the other systematic reviews, therefore represented an opportunistic sample for conducting the current small case study. These sources were considered to offer useful evidence on the data extraction process because they each represent a high-impact, highly accessed source of systematic reviews. A fourth relevant review was identified [7], but does not form a key part of this evaluation because it is published in a relatively low-impact journal in Chinese and does not include all of the studies or outcomes. It is therefore referred to only where relevant.

An analysis was made of certain binary event data extracted for the following three key outcomes, common to all three reviews: dislocation rates; one-year mortality rates; and revision rates. These outcomes were all relatively straightforward. Other outcomes, such as “reoperation” or “any surgery”, which could include dislocations and other alternative procedures were not used as they presented obvious problems of definition. The data for analysis were derived from the publications of those trials common to all three reviews. Data on the three stated outcomes were independently extracted from the included studies by the three authors of this paper, and any discrepancies or disagreements were discussed and consensus met, to create the “HTA” data that acted as the “reference standard”. This term is not intended as a judgment of quality, but rather simply represents the baseline that was required for such an evaluation. We chose to use our own HTA report as the nominal reference standard for the comparisons, despite being the most recent review, because it contained the data we, as a group, had carefully selected and agreed. However, in many ways, the choice of reference standard did not matter; the principal outcome of interest was to identify instances of inconsistency across the reviews, which then needed to be explored with reference to the original, primary studies. In reality, these provided the real reference standard.

The data for analysis therefore consisted of the denominators and numerators for each outcome across two trial arms (4 variables) in six studies common to each review. This gave a total of 24 variables for each outcome, which could be the same or different for each review. The results presented below describe the percentage of differences between the BMJ or Cochrane review and the HTA “reference standard” for any of the outcomes. Thus, if six of the variables were different between, for example, the BMJ and HTA reviews, that represented a 25% (6/24) difference. An investigation was then made to determine whether alternative data existed, which might explain the differences, and for which reviewers had been required to make an explicit choice. Such differences were categorised as “selection”. In cases where the data were clearly incorrect, that is, the data extracted by a review did not reflect any of the potential data reported in the original primary studies, these were categorised as “errors”. Percentages are reported for each category. The impact of data differences on the summary estimates of effect using relative risks (RR) was also calculated by meta-analysis using a random effects model.

## Results

A comparison of the three reviews’ extracted data for each outcome and each relevant trial is shown in Tables 1, 2 and 3. The likely explanations of selections or errors are given in the final column of each table.

**Table 1:** Dislocation outcome data reported for THA (n/N) vs HA (n/N) in 3 reviews

**Study**	**BMJ**	**Cochrane**	**HTA**	**Comments**
Dorr 1986 [22]	7/39 vs 2/50	7/39 vs 2/50	7/39 vs 2/50	Identical
Skinner 1989 [17]	NR	10/80 vs 11/100	11/89 vs 10*/91	Skinner and Ravikumar only report percentages rather than event data and only Ravikumar reports numbers in each trial arm (denominator)
(1-year data)				
				**Errors:**
Ravikumar 2000 [18]	18/91 vs 12/89	NR	18/89 vs 12/91	BMJ denominators for the 2 groups are the wrong way round; Cochrane generates its own denominators having failed to identify Ravikumar 2000 (follow-up to Skinner)
(13-year data)				
				**Selection difference:**
				Numerators are all incorrect due to calculations based on percentages and incorrect denominators.
Baker 2006 [12]	3/40 vs 0/41	3/40 vs 0/41	3/40 vs 0/41	Identical
Keating 2006 [11]	3/69 vs 3/111	3/69 vs 2/69	3/69 vs 2/69	**Selection difference:**
				BMJ alone analyses HA data from a separate trial arm (with 111 participants), but these data from this arm arguably should not be included in this analysis because different eligibility criteria were being applied (i.e. the surgeons and centres involved were either unwilling or unable to have participants randomised to THA).
Blomfeldt 2007 [9]	0/60 vs 0/60	0/60 vs 0/60	0/60vs 0/60	Identical
Macaulay 2008 [23]	1/17 vs 0/23	1/17 vs 0/23	1/17 vs 0/23	Identical
Mouzopoulos 2008 [8]	NR	NR	NR	NA
	**2/24 errors =** **8%**	**2/24 errors =** **8%**		**6 analysed studies =** **a/B vs c/D =** **24 variables**
	**4/24 selection differences =17%**	**2/24 selection differences =8%**		

*Liang has 8/91 here: an error. Otherwise Liang has the same data as the HTA. *THA*, Total Hip Arthroplasty; *HA*, Hemiarthroplasty; *NR*, Not Reported; *NA*, Not Applicable. Findings are given in bold.

**Table 2:** Mortality data at 1 year reported for THA (n/N) vs HA (n/N) in 3 reviews

**Study**	**BMJ**	**Cochrane**	**HTA**	**Comments**
Dorr 1986 [22]	3/39 vs 4/50	NR	NA (event data for each arm NR)	**Error:**
				Dorr reports 7 deaths across both arms; BMJ review categorises this as 3 and 4 in each arm [10]
Skinner 1989 [17]	21/91 vs 24/89	18/80 vs 27/100	20/89 vs 25/91	**Error** and **Selection difference:**
(1-year data)				As Table 1 for these data
Ravikumar 2000 [18]				
(13-year data)				
Baker 2006 [12]	NR	NR	NR	NA
Keating 2006 [11]	4/69 vs 11/111	4/69 vs 6/69	4*/69 vs 6/69	**Selection difference:**
				As Table 1
Blomfeldt 2007 [9]	4/60 vs 3/60	4/60 vs 3/60	4/60 vs 3/60	Identical
Macaulay 2008 [23]	1/17 vs 5/23	NR	NR	**Selection difference:**
				BMJ analyses reported 6-month data as 1 year data; Cochrane, HTA and Liang [7] all omit it from the 1-year analysis; Cochrane analysis uses these data for 6-month follow-up only. It is unclear when the additional deaths reported for up to 2 years (4/17 vs 7/23) occurred. They may have occurred in the 6–12 month period.
Mouzopoulos 2008 [8]	10/43 vs 13/43	6/43 vs 6/43	6/39 vs 6/38	**Selection difference:**
				Denominators: HTA applied an intention-to-treat analysis, excluding 4 from THA arm (2 data lost; 2 not satisfy inclusion criteria) and 5 from HA arm (none satisfied trial’s inclusion criteria), as they did not satisfy the study’s inclusion criteria and no follow-up data were collected. [13] If these are included, then explicit best and worst case analyses perhaps should have been performed, with imputations explained [14]
	**4/24 errors =** **17%**	**2/16 errors =** **13%**		**4-6 analysed studies =** **a/B vs c/D =** **16 or 24 variables:**
	**10/24 selection differences = 42%**	**4/16 selection differences = 25%**		

*Liang has 5/69 here: an error. Otherwise Liang has the same data as the HTA. *THA*,Total Hip Arthroplasty; *HA*, Hemiarthroplasty; *NR*, Not Reported; *NA*, Not Applicable. Findings are given in bold.

**Table 3:** Revision rates outcome data reported for THA (n/N) vs HA (n/N) in 3 reviews

**Study**	**BMJ**	Cochrane*	**HTA**	**Comments**
Dorr 1986 [22]	2/39 vs 4/50	2/39 vs 4/50	2/39 vs 4/50	Identical
Skinner 1989 [17]	NR	3/80 vs 13/100	4/89 vs 12/91	As Table 1 above, plus an additional **error:** BMJ calculates 25% rather than the reported 24% for numerator
(1-year data)				
Ravikumar 2000 [18]	6/91 vs 22/89	NR	6/89 vs 22/91	
(13-year data)				
Baker 2006 [12]	1/40 vs 6/41	1/40 vs 3/41	1/40 vs 6/41	**Selection difference:**
				BMJ, HTA and Liang [7] all report 1/40 vs 6/41; Cochrane omits 3/41 which were classified as planned or awaiting revision; the event had not taken place but was “planned” only, so was not counted
Keating 2006 [11]	NR	NR	NR	Identical
Blomfeldt 2007 [9]	4/60 vs 3/60	1/60 vs 0/60	0/60 vs 0/60	**Selection difference:**
				Cochrane 1/60 is a “revision” described by Blomfeldt as a “wound revision”; only revision of implant counts as a revision in the HTA; the BMJ review figures include re-operations both on the contra-lateral side, not related to the implant, and for trauma of the lower limb. [10]
Macaulay 2008 [23]	1/17 vs 0/23	1/17 vs 0/23	1/17 vs 0/23	Identical
Mouzopoulos 2008 [8]	1/43 vs 3/43	1/43 vs 5/43	1/39 vs 5/38	**Selection difference:**
				Numerators: BMJ “excluded” 2 HA revisions from the analysis (so 3 rather than 5) but this was arguably not justified as the individuals had the outcome of interest and so should have been included;
				Denominators: As Table 2.
	**3/24 errors =** **13%**	**2/24 errors =** **8%**		**6 analysed studies =** **a/B vs c/D =** **24 variables**
	**6/24 selection differences = 25%**	**5/24 selection differences = 21%**		

*Revisions in Cochrane are categorised as “Major reoperations”. Findings are given in bold.

For the dislocation outcome, the percentage of differences due to the selection of alternative but potentially valid data were 17% and 8% in the BMJ and Cochrane respectively, where-as 8% were categorised as “errors” in both reviews (2/24); (see Table 1). For the 1-year mortality outcome, compared to the HTA report, the percentage of different data due to differences in selection was 42% and 25% in the BMJ and Cochrane reviews respectively, while differences categorised as “errors” were 17% and 13% (see Table 2). For the revision rates outcome, 21% and 25% of data differences in the BMJ and Cochrane reviews, respectively, compared to the HTA report, were due to selection, and 13% and 8% of different data were categorised as “errors” (see Table 3).

The reasons for the differences between data could often be easily ascertained and are described in the final columns of Tables 1, 2 and 3. The explanation of those differences categorised as “errors” was straightforward. They were due either to errors in transposing data from primary studies for analysis, or a failure to identify the correct data from the available publications. In this latter instance, a best guess seems to have been used. However, it was not always possible to see the rationale behind the selection of other data, such as the 1-year mortality or revisions data used in the BMJ review and extracted from the trials reported by Mouzopoulos et al. [8] and Blomfeldt et al. [9], respectively. This was only clarified by gaining a response from the authors [10]. The differences in the extracted data did lead to small differences in meta-analysed relative risks between the two treatments in the three reviews, but none was significant. See Table 4.

**Table 4:** **Risk of outcomes for THA vs HA using the data reported in the 3 reviews (RR, 95**% **CI), p value**

**Outcome**	**HTA**	**Cochrane**	**BMJ**
Dislocation	1 year: 1.70 (0.91, 3.19), p = 0.10	1 year: 1.71 (0.91, 3.21), p = 0.10	
	13 years: 1.93 (1.10, 3.37), p = 0.02		13 years: 1.88 (1.08, 3.26), p = 0.03
Mortality at 1 year	0.85 (0.57, 1.29), p = 0.45	0.87 (0.57, 1.32), p = 0.51	0.80 (0.56, 1.14), p = 0.22
Required revision	1 year: 0.38 (0.18, 0.81), p = 0.01	1 year: 0.40 (0.18, 0.89), p = 0.02	
	13 years: 0.33 (0.17, 0.64), p = 0.001		13 years: 0.47 (0.22, 1.01)*, p = 0.05

A random effects model was used for all analyses. Statistical heterogeneity of I^2^ = 0% for all analyses, except where specified.

*I^2^ = 23%; *THA*, Total Hip Arthroplasty; *HA*, Hemiarthroplasty; *RR*, Risk ratio; *CI*, Confidence Interval.

## Discussion

Differences in data extracted due to selection were much more frequent than differences due to errors. In each case, a choice existed between various relevant outcome data, so the reviewers had to make a decision about which data to extract and use. However, the BMJ and Cochrane reviews did not always make explicit the reasons for their particular choice of data. For example, the BMJ review chose to include data from an alternative arm in one trial [11] and to include data relating to both hips, rather than just the hip receiving the index procedure, for the revision outcome in another trial [9]. In both instances, this led to the review analysing data that were different from the Cochrane review and the authors’ own HTA report, which were identical. Alternatively, both the BMJ and HTA reviews aggregated the reported “planned” as well as “completed” revisions data for the study by Baker et al. [12] (this was explicitly reported by the HTA review), while the Cochrane review only extracted and analysed the former. The HTA review explicitly excluded participants from the 1-mortality data for Mouzopoulos et al. [8] because the authors felt they should not have been randomised, due to an implementation error [13], while the Cochrane and BMJ reviews both included these participants. Good arguments could be made for any of these choices. However, some selections might be deemed rather more questionable. Examples of this include the BMJ review’s inclusion of revisions and exclusions as deaths, without imputation for these missing data [14], and excessive extrapolation (6-month data as 1 year data) (see Table 3 and [10]). In none of the above instances of selection did the Cochrane or BMJ reviews justify their selection decisions.

This is not a criticism of these reviews, but rather reflects a problem that is arguably endemic in published examples of the systematic review method. After all, if reviewers are making what they consider to be a reasonable choice, then they arguably would not feel the need to record this. The data might seem the only reasonable data to extract. However, the lack of transparency over the data being extracted and analysed in reviews has been raised as an issue before [3] and it is our contention that, as reviewers, we are expected to be explicit and transparent in our actions and choices. The GRADE group have recently called for reviewers to provide greater clarity in their decisions regarding assessments of risk of bias: reviewers should make explicit their reasons for a particular judgement. [15] We feel that an appeal for similar clarity is required for the data extraction process. The recording of a decision, when one has been made, should form a part of both the process and the reporting of systematic reviews.

The incidence of errors appears to be low based on this small case study sample. They appear to occur principally due to human error, although double data extraction should minimise the chance of such bias or random error [16]. Both the BMJ and Cochrane reviews reported using this approach, and the number of errors was indeed relatively small, usually no more than 8% of the data for any of the three outcomes assessed. The majority of these instances related to one study [17,18] and were either transposition errors when inputting the data into statistical software [10] or were errors in calculating review data from primary study data: the outcomes in the primary study publication were only reported as percentages and the numbers in each arm were only reported in one of the two papers reporting the study (and this second paper was missed by one of the reviews). Errors of transposition and calculation have been identified as a problem previously [3,19], although it has also been pointed-out that these should not occur if at least two reviewers are involved [3]. The relatively small number of errors also fits with previous findings that error rates are lower when the variable is “simple”, such as here. [1,2] Also, in each of these instances the impact on the actual overall results was very small because the proportion of events was often correct (a key factor in the analysis), even if the actual numerators and denominators extracted were incorrect.

Double data extraction is the obvious facilitator here and is the recommended method [20]. This is because it not only controls for random error, but can also highlight the presence of data choices since, where a choice exists, the scope for variation in extraction is higher. In the context of completing reviews to time and cost [21], where double-data extraction might not be feasible, “independent verification” [1] or double-checking of key outcome data by a second reviewer might offer a more rapid and pragmatic approach. While double data extraction can be very time-consuming and has actually been found not to produce large or significant differences in comparison with single data extraction [2], the simple checking of data for analysis alone may represent a reasonable way forward.

Finally, the journal peer-review process should perhaps also involve some scrutiny of extraction to determine if errors or selection has occurred. Currently, peer reviewers accept the data as given. The introduction of a process of double-checking for at least of sample of the extracted data in any review submitted for publication may produce a Hawthorne effect: errors might be avoided and selections justified if authors know their work is to be scrutinised. However, single extraction has been shown to produce errors even in specific pieces of research testing for data extraction error [1,2]. While the assessments of data extraction published so far have admittedly found that many extraction errors make little difference to bottom-line outcomes, this is no reason not to do undertake such processes in review and peer-review. After all, something is always better if it is true, or if it at least allows readers to judge a review’s decisions for themselves.

The principal limitation of this research is that is an opportunistic small study, which has only compared three reviews and their binary event data. A much larger study or a study of a different topic might find that the frequency of both errors and differences of selection is lower. Alternatively, a comparison of a topic with more complex outcomes might produce even higher rates of difference in error or selection: an assessment of data extracted to calculate standardised mean differences (SMD) identified frequent and sometimes large errors [3]. The findings of this present study are not generalisable to all reviews, but serve to highlight the issue of interpretation and selection that potentially affects data extraction and, consequently, analysis in standard effectiveness reviews using quantitative methods. This problem has otherwise only been commented on in a single study [3]; the present case study extends that work and illuminates the issue further. However, more research on this topic is needed.

## Conclusion

Data extraction is not a straightforward objective process without complexity. The capacity for errors to occur is well known, while there has been comparatively little recognition of the capacity of reviewers to make different but equally justifiable choices in data selection. These are not “errors” as such, but they are certainly a fact of conducting reviews. Such choices can affect a review’s results. In cases where some of the event data being analysed by a review might be viewed as being open to question, i.e. a matter of selection or interpretation, reviewers should make every effort to clarify or explain their choice of outcome data, within the scope of their publication. Double data extraction can help control both for errors and to identify issues of selection. Systematic review was developed as a method specifically to be reproducible and transparent. The integrity and robustness of the method can only benefit from the full application of such processes.

## Competing interests

The authors declare that they have no competing interests.

## Authors’ contributions

CC conceived and designed the case study; CC, AS and EK extracted and interpreted the data. CC drafted the paper, and AS and EK undertook critical revision of important content of the manuscript. All authors approved the final version of the manuscript.
